# Testing of Homogeneity of Coordinates of Various Permanent GNSS Reference Stations Networks of the Republic of Serbia According to the Common Requirements for Proving Competence

**DOI:** 10.3390/s22207867

**Published:** 2022-10-16

**Authors:** Jelena Gučević, Olivera Vasović Šimšić, Siniša Delčev, Miroslav Kuburić

**Affiliations:** 1 Faculty of Civil Engineering Subotica, University of Novi Sad, 24000 Subotica, Serbia; 2 Department School of Civil Engineering and Geodesy of Applied Studies, Academy of Technical and Art Applied Studies Belgrade, 11000 Belgrade, Serbia

**Keywords:** permanent GNSS stations network, GNSS network comparison, GNSS RTK measurements, metrological uncertainty, metrological traceability, assigned value, requirements for competence and performance, sources of uncertainty

## Abstract

The validity of the results obtained within different permanent GNSS reference station networks (GNSS Network) must be periodically controlled using criteria that are generally known from statistical analyzes or prescribed by International Standards. Procedures for evaluating the uncertainty of measurements are defined in accordance with the purpose of the GNSS Network. The authors of this paper want to point out the need to establish requirements for periodical and systematical control of GNSS coordinates within the same permanent GNSS Network and control of GNSS coordinates between different permanent GNSS Networks measured on the same/unique point on the ground. This paper presents control procedures for three permanent GNSS reference station Networks established and operating in the Republic of Serbia. Special attention is on the analysis of data consistency within one permanent GNSS Network and the mutual consistency of GNSS data between different networks. The paper aims to promote reliance on the different GNSS Networks and contains suggestions on how GNSS Networks may prove that they are performing competently and that they can provide valid results for field measurements. Particularly highlighted is the need to plan and implement measures related to increasing the effectiveness of the GNSS system, achieving improved results, and preventing negative effects while performing field measurements. The paper presents the results for comparison, selected according to the rules for creating a Digital Cadastral Map features, i.e., points, lines, and polygon. The results for comparing point features are the GNSS coordinates. The results for comparing line features are the lengths of the line, i.e., distances, and the results for comparing polygon features are the areas of the polygons.

## 1. Introduction

The Global Navigation Satellite System (GNSS) positioning problem is set upon: “(i) utilization of the common spatial reference coordinate system (World Geodetic System 84 (WGS84)), (ii) utilization of the common time frame (Universal Time Coordinated (UTC)), (iii) satellite signals propagation at the constant velocity equal to the velocity of light in vacuum” [1,2]. The efficiency of positioning in the navigation part of the GNSS Network is analyzed using a methodology that includes the GNSS observations collection with the consumer-grade smartphone GNSS receivers [3]. The efficiency of GNSS positioning for geodetic purposes and its class of GNSS coordinate accuracy, can be compared and analyzed, only when the measurements are performed at the same location, under the same measurement conditions, and using the same GNSS receivers and their auxiliary equipment and software for data processing.

The permanent GNSS reference stations network consists of a set of reference points equipped with permanently operating GNSS receivers and antennas, which together with the control center, necessary hardware, software, and communication infrastructure enable the collection, archiving, processing, modeling and distribution of GNSS data [4].

The number of GNSS Networks is increasing worldwide and they tend to be integrated into a GNSS system that includes one country, several neighboring countries or an entire continent. GNSS Networks provide useful datasets to facilitate the investigations of various geophysical phenomena, such as global plate motion, regional tectonic deformation, uplift of the Plateau, land subsidence, environmental loading effects, hydrological cycle [5]. GNSS permanent reference stations established on the European continent tend to be integrated into the EUREF (European Reference Frame) Permanent Network—EPN [6]. “The primary purpose of the EPN is to provide access to the European Terrestrial Reference System 89 (ETRS89) which is the standard precise GNSS coordinate system throughout Europe.” [7]. “The EPN contributes also to the monitoring of tectonic deformations in Europe, and supports long-term climate monitoring, numerical weather prediction and the monitoring of sea-level variations.” [7].

According to the principle of territorial integration the Crustal Movement Observation Network of China (CMONOC) was developed, whose data are shared by numerous institutions. CMONOC has a wide range of applications in diverse areas with high precision and high spatial resolution [8]. “Analyzing the GPS position time series, a long-running, continental-scale, and high-density GPS network, can provide a wealth of information on the periodic signals, noise characteristics, and sources of the common mode component (CMC) for GPS position time series, which can be a reference for similar studies over other regions.” [5]. “Comprehensive understanding of the nature and characteristics of the nonlinear variations of GPS height time series is important to the investigations of geophysical phenomena, the analysis of GPS error sources, and the modeling for GPS height time series.” [9].

The first GNSS Network on the territory of the Republic of Serbia that was established and was primarily used for the purposes of performing geodetic works within the scope of works of the Republic Geodetic Authority (RGA) is the AGROS (Active Geodetic Reference Network of Serbia). RGA is a special government organization performing professional and public administration works pertaining to a state survey, real estate cadastre, utility cadastre, basic geodetic works, address registry, topographic-cartographic activities, property valuation, geodetic-cadastral information system, National Spatial Data Infrastructure (NSDI) and geodetic works in engineering-technical fields in the Republic of Serbia.

The AGROS covers the whole territory of the Republic of Serbia with GNSS services. The network was established during the period from 2002 until 5 December 2005, while economic use of the AGROS service began on 16 December 2005. Ref. [10] At that time, the RGA had issued Professional instructions for the application of the AGROS within Basic Geodetic Works and Real Estate Survey.

In April 2011 was carried out an integration of three AGROS GNSS permanent reference stations named: SABA00SRB; KNJA00SRB and NPAZ00SRB into the EPN [11]. Those three GNSS permanent reference stations became part of the International Terrestrial Reference System (ITRS), i.e., its realization in the year 2008 (ITRF2008). In this way, RGA was ranked as one of the international institutions that deal with the analysis of GNSS data.

Until 2018, the AGROS was the only system of permanent GNSS reference stations on the territory of the Republic of Serbia that could be used for the purposes of performing geodetic works within the scope of works of the RGA [12].

“In the Bernese GNSS software, there are modules developed for quality control of AGROS reference station point coordinates, evaluation of tropospheric and ionosphere refraction parameters, calculation of hourly deviations of terrestrial Continuously Operating Reference Stations (CORS) receivers and Global Positioning System (GPS) satellites, as well as for cumulative multi-year calculation of tectonic displacements of GNSS reference points.” [13]. AGROS is performing daily GNSS data processing. These results are publicly available for AGROS users at the AGROS control center Web portal [10].

In October 2018, the Republic of Serbia adopted the Rulebook on the establishment of the permanent GNSS reference stations networks which regulates geodetic works in the process of designing and implementing the permanent GNSS Network; defines the content of the measurement report on the determination of GNSS coordinates, as well as the performed quality control of GNSS permanent stations network reference points coordinates in the National Coordinate System. The National Coordinate System for the Republic of Serbia is European Terrestrial Reference Frame 2000 (ETRF2000).

Over time, the market for goods and services in the geodetic profession recognizes the possibilities of investing in GNSS technology, both locally and globally. Locally, the increasing numbers of private geodetic organizations in the Republic of Serbia are equipped with a GNSS receiver for their needs in geodetic surveying. Globally, on part of the territory or on the entire territory of the Republic of Serbia, a privately owned permanent GNSS reference stations networks are starting to be established, and in October 2018, the RGA issued a Rulebook for the establishment of the permanent GNSS reference stations networks (Rulebook).

### 1.1. The Permanent GNSS Reference Stations Networks in the Republic of Serbia

The establishment of the GNSS Networks on the territory of the Republic of Serbia is carried out according to the Rulebook [4].

The Rulebook stipulates that the Terms of Reference on the establishment of the GNSS Network are provided by its Investor and submitted to the RGA for approval and verification. The GNSS Network can only be established by a geodetic organization (i.e., Investor) that has the appropriate license. The geodetic organization must have the Certificate of Origin for the equipment and software used for the establishment of the GNSS Network. The GNSS Network should consist of at least six permanent stations (i.e., reference points), which are distributed approximately evenly over the area they cover with a maximum distance of up to 70 km among them. The geodetic organization that has established a GNSS permanent stations network must provide full access for supervision of the operations of its GNSS Network Control center to RGA. Professional supervision over the establishment of a private GNSS Network is carried out by the RGA.

The Technical report on the establishment of a GNSS Network must contain: the disposition of selected locations for the permanent GNSS reference stations point placement with a description of their position and method of stabilization; technical specification of installed GNSS equipment with the Certificate of Origin and Metrological Certificate of the correctness of the measuring instrument; technical specification of equipment and software in the GNSS Network Control center; report on determining the coordinates of GNSS permanent stations reference points in the National Coordinate System (ETRF2000); report on the implemented quality control of established GNSS permanent stations. All the above reports refer to the quality of the GNSS coordinates of the GNSS Network, starting from: reconnaissance, stabilization, measurements, and data processing, up to the calculation of ground point coordinates [4].

Control of functionality of the newly established GNSS Network is carried out by control positioning measurements at five reference points of the SREF network (SREF—Geodetic Reference Basis of Serbia [14]), evenly distributed over the area covered by the GNSS Network. During control positioning measurements, the GNSS receiver is placed on a tripod or stick with a bipod. GNSS observations are made with at least three measurements lasting 30 s each, with a registration interval of one second. Definitive coordinates of control SREF points are calculated as the mean of three GNSS measurements, provided that the range between the minimum and maximum values of the coordinates is less than 20 mm in horizontal position and 30 mm in height. The procedure and results of quality control measurements are documented in the technical report on the establishment of a GNSS permanent station network. In this manner, the functionality of the GNSS Network is proven. The RGA carries out professional supervision and when it determines that the works have been carried out according to the technical documentation and in accordance with the regulations and quality standards, RGA issues a decision on the establishment of the GNSS Network. The GNSS network is then officially approved for use for the purposes of performing geodetic works within the scope of works of the RGA.

**Figure 1:**
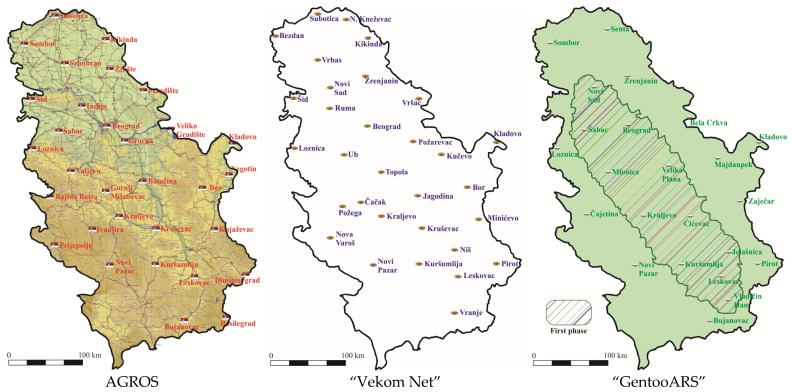
GNSS Networks in the Republic of Serbia.

To date, the RGA has confirmed two privately owned permanent GNSS reference stations networks (Figure 1):

- The “Vekom Net” GNSS Network which is since 2018 an integral part of the State Spatial Reference Framework of the Republic of Serbia and can be used for the purposes of performing works within the scope of works of the RGA. The “Vekom Net” is available on the entire territory of the Republic of Serbia [15].
- “GentooARS” (first phase) GNSS Network was established for the part of the territory of the Republic of Serbia and has 10 permanent GNSS reference station points. In 2019, it was approved for official use for an area that includes 2529 cadastral municipalities in the Republic of Serbia [16].
- The authors of this paper did not have access to the information about the methods of calculating corrections of GNSS measurements applied by GNSS Networks: “Vekom-Net”; “GentooARS”.

### 1.2. Reasons for Initiating the Present Research

In geodetic practice, the field procedures for testing GNSS field measurement system in real-time kinematics (GNSS RTK) and their ancillary equipment are performed according to the procedures defined by the ISO 17123-8 Standard [17]. So far, the Accreditation Body of Serbia (ABS) in the Republic of Serbia has accredited three laboratories [18,19,20] according to the ISO/IEC 17025:2017 [21] Standard. Those laboratories in their Scope of Accreditation use the reference document ISO 17123-8. Accredited laboratories perform interlaboratory comparisons (ILC) in accordance with the ISO 17043 standard [22] and check the measurement uncertainty of the GNSS RTK system (GNSS receiver and antenna) and their ancillary equipment as per their Scope of Accreditation. The measurement uncertainty is calculated and presented according to the Guidelines given in document EA-4/02 M [23].

However, continuous testing of the operation of the GNSS Networks in use on the territory of the Republic of Serbia is still not required. The need for this testing arose from the knowledge, experience, and situations that were noted during the calibration of the GNSS systems in the accredited laboratory No. 02-077 [20]. Specifically, in January 2022, laboratory No. 02-077, in which two of the authors of this paper participate, recorded deviations during the calibration of the GNSS system. To confirm their assumptions, a measurement was planned and carried out with the GNSS system: SOKKIA—GRS 2700ISX; software: Carlson SurvCE with defined parameters for measurement monitoring and control: Horizontal Standard Deviation (HSDV): 0.020 m; Vertical Standard Deviation (VSDV): 0.030 m. Measurements were performed for three consecutive days at two ground points that belong to the laboratory’s testing polygon in laboratory No. 02-077. The measurements consisted of three series in the AGROS network. The first series included 5 GNSS RTK measurements alternately at point 1 and point 2 of the testing polygon; after a pause of 15 min the second series of five GNSS RTK measurements were performed alternately at points 1 and 2; after another pause of 15 min, the third series of measurements was performed. In total, 30 values were registered in one day (15 measurements at point 1 and 15 measurements at point 2). For each day of measurement, deviations from the mean value were calculated by coordinates (*E*, *N*, Δ*h*) in the European Terrestrial Reference System 1989/Universal Transverse Mercator (ETRS89/UTM) system zone 34N system.

Table 1 shows the characteristic deviation of GNSS coordinate values for each day of measurement. The registered deviations from the mean value on the third day (0.400 m in position and −0.644 m in height) were sufficient reasons to contact the administration of the AGROS and inform them about the discrepancies in values. The administration of the AGROS network was grateful for the information received and proceeded to resolve the issues reported. At the same time, the administrators of the “Vekom Net” and “GentooARS” networks were informed about the observed deviations and possible problems. The same information reached the professional users of GNSS Networks in order to prevent possible consequences of bad measurement results and their further processing and application. The administrators of all three GNSS Networks have expressed their interest in designing and conducting an InterGNSS Network comparison within the framework of different GNSS Networks in cooperation with the authors of this paper.

From this examination and the results obtained, it can be concluded that IntraGNSS Network comparison should be planned and successively performed for each GNSS Network. IntraGNSS Network comparison should be performed especially when expanding and improving hardware and software systems. In order for the GNSS system (which counts three GNSS Networks) to be completely reliable at the level of the Republic of Serbia, it is necessary to plan an InterGNSS Network comparison in accordance with ISO 17043.

## 2. Research Objective and Methods

The procedure by which, under certain conditions, the relationship between declared values and values with obtained associated measurement uncertainties and provided by approved measurement methods is called calibration. “…calibration of measuring devices are traditional tasks of all metrological oriented engineers. The necessity for this is justified in the fact that all measuring processes are influenced as well by random as by systematic errors. Also, surveyors have the task and duty to report reliable measurement results and reasonable accuracy data…” [24]. The first edition of the ISO 17123-8 standard was published in 2007 [24]. This standard envisages testing the GNSS RTK system during field measurements, does not require additional software for processing, and data testing is based on elementary statistics [19]. However, in countries where a large number of GNSS Networks are in official use, the functionality of the GNSS RTK system should be checked through:

- IntraGNSS Network comparison, within the same GNSS Network,
- InterGNSS Network comparison, within diverse GNSS Networks.

IntraGNSS Network comparison involves organizing, performing, and evaluating measurements with different GNSS RTK systems within the same GNSS Network, in accordance with pre-determined conditions. IntraGNSS Network comparison can be performed according to the procedures defined by the ISO 17123-8 standard. These procedures are intended to be field checks when GNSS receiver, antenna, and their ancillary equipment are used in building, surveying and industrial measurements.

InterGNSS Network comparison involves organizing, performing, and evaluating measurements carried out with the same GNSS RTK systems and within different GNSS Networks, in accordance with predetermined conditions. InterGNSS Network comparison can be performed according to the procedures for proving competence. Those procedures are defined by the ISO 17043 standard [22].

### 2.1. IntraGNSS Network Comparison

The International Federation of Surveyors (FIG) has foreseen a model presented in 4 stages for testing of geodetic equipment [25]:

- The first phase (I) is a simple functional check that evaluates operability, visual inspections of the equipment, and performs measurements in the field in short intervals;
- In the second phase (II), in the extended functionality check, under which significant deviations of individual measurement results at regular intervals or event dependence (i.e., cyclical repetition of an individual impact) are evaluated;
- The third phase (III) represents calibration, where GNSS observations are compared with their reference (nominal) values; and
- The fourth phase (IV) refers to the determination of the technical characteristics of the instruments with the aim of obtaining a certificate for each instrument.

Phases I, II, and III should be used during field testing of the GNSS RTK systems for a single location, but should not be equalized with ISO 17123-8 calibration (phase IV). Certification for each instrument is carried out in laboratories accredited by the National Accreditation Body. A geodetic organization, which is an investor in the establishment of the GNSS Network, should perform a check of the system functioning by direct GNSS field measurements and control of the coordinates (comparison) obtained within its GNSS Network (phases I, II, and III). This control should be done for its users, when granting them access and use of the GNSS Network. The comparison should be made for coordinates in the national coordinate system of the Republic of Serbia (ETRF2000). When using a GNSS Network, the base point is equated to a network reference point. The rover points should be placed between 2 m and 20 m apart. The rover points represent the field calibration polygon. The horizontal distance and height difference between polygon points ($S_{i,j}$ and $\Delta h_{i,j}$) are provided from measurements that are more accurate than the GNSS RTK method.

The IntraGNSS Network comparison involves measurement according to the “Simplified test” procedure. This procedure implies that five sets of GNSS coordinate measurements should be obtained in the ETRF2000 system. Individual measurements are directly compared to nominal values in order to detect each measurement with a gross error. The reference values of the coordinates ($X_{1,2}^{N},\;Y_{1,2}^{N},\;h_{1,2}^{N}$) should be determined in the process of establishment of the GNSS Network, i.e., $S^{N}$ can be calculated. From the field measured coordinates ($x_{1,2}^{},\;y_{1,2}^{},\;h_{1,2}^{}$), (*j* = 1, …, 5) in the series (*i* = 1), the following can be calculated:

(1) $$\begin{array}{l} s_{i,j}^{}=\sqrt{{\left(x_{i,j,2}-x_{i,j,1}\right)}^{2}+{\left(y_{i,j,2}-y_{i,j,1}\right)}^{2}}\\ \Delta h_{i,j}^{}=h_{i,j,2}-h_{i,j,1} \end{array}$$

where:

$s_{i,j}$ and $\Delta h_{i,j}$ are calculated horizontal distance and height difference respectively in the set *j* in series *i*.

Statistical testing involves calculating the deviation of the horizontal distance and height difference respectively ($\varepsilon_{S}{}_{i,j}^{}$, $\varepsilon_{\Delta h}{}_{i,j}^{}$) as follows:

(2) $$\begin{array}{l} \varepsilon_{S}{}_{i,j}^{}=s_{i,j}^{}-S^{N}\\ \varepsilon_{\Delta h}{}_{i,j}^{}=\Delta h_{i,j}^{}-\Delta h^{N} \end{array}$$

If the deviations do not meet the following conditions:

(3) $$\begin{array}{l} \left|\varepsilon_{S}{}_{i,j}\right|\leq 2.5\cdot \sqrt{2}\cdot \sigma_{xy}\\ \left|\varepsilon_{\Delta h}{}_{i,j}\right|\leq 2.5\cdot \sqrt{2}\cdot \sigma_{h} \end{array}$$

where $\sigma_{xy}$ and $\sigma_{h}$ are values specified by the manufacturer of the GNSS equipment, then the measurements/test procedure should be repeated and the cause for the deviation discovered. The functionality of individual components of the GNSS RTK equipment, both hardware and software, is not known in detail to its user, and this test should verify the operability of the GNSS Network.

After assigning a username to the future user of the GNSS Network, the user of the GNSS RTK equipment checks the technical characteristics of the GNSS receiver and antenna in order to obtain a Certificate of Calibration. Calibration of GNSS equipment in the Republic of Serbia can be performed in one of three accredited metrology laboratories [18,19,20] and is not the subject of this paper.

### 2.2. InterGNSS Network Comparison

Proper functionality and accordance with the prescribed parameters of a GNSS network is confirmed by issuing the certificate for official use; however, a permanent InterGNSS Network comparison would ensure an independent control of the quality of the observations’ results (points’ coordinates). The quality of performance of a GNSS Network is essential for its users and their clients that use data acquired in the GNSS RTK mode. For measurement results (coordinates of points) their metrological traceability should be confirmed.

“Metrological traceability, property of a measurement result whereby the result can be related to a reference through a documented unbroken chain of calibrations, each contributing to the measurement uncertainty” [26]. Specifically, for the operational GNSS Networks, in the process of metrological traceability, each result that contributes to the measurement uncertainty would be checked independently of the GNSS Network in which it was measured. This would facilitate the comparison of measurement results (coordinates), confirmation of the achieved uncertainty in measurement, identification of problems in the operation of the GNSS Network, initiating action for its improvement, and provide additional confidence in the GNSS Network to its users.

InterGNSS Network comparison includes: selection of the assigned value; test scheme planning; defining GNSS equipment handling; carrying out GNSS measurements to determine stability, homogeneity, and uncertainty in measurement; performing statistical analysis; evaluating the results achieved participating GNSS Networks (further on “Participants”) and providing a test report. InterGNSS Network comparison should be performed in accordance with the guidelines from the ISO 17043 Standard [22]. The Standard contains general requirements for competence and performance evaluation of participants according to pre-established criteria.

There are different methods for determining the assigned value and its uncertainty. Assigned values are designated as reference values and must be in accordance with the objectives of control and confirmation of competence. Reference values can be determined using any of several procedures described in the ISO 17043 standard: “B.3. Determination of the assigned value and its uncertainty” [22]. In this particular case, it is possible to use the procedure of “formulation values”, which involves the preparation of a comparison and the selection of reference values from the primary GNSS Network, or to determine the assigned value and its uncertainty in the static GNSS positioning mode.

The results for comparison are transformed into bias *D*, score *z* and number *E*, which is in accordance with the standard for evaluating the performance of participants (ISO 17043: “B.4. Calculation of performance statistics”) [22]. The results are designated as “Satisfactory” or “Unsatisfactory”.

Common statistical approaches are:

(a) **bias** *D*, when the absolute difference is formed [27] and can be calculated as follows:

(4) D=(x−X)

or as a percentage $D=\frac{\left(x-X\right)}{X}\cdot 100\%$, where:

*X* is the assigned value,

*x* is the result from Participant.

The following statistics are used to interpret the results:

(5) $$2\cdot \hat{\sigma }<D<-2\cdot \hat{\sigma }$$

(6) $$3\cdot \hat{\sigma }<D<-3\cdot \hat{\sigma }$$

where $\overset{⌢}{\sigma }$ is the standard deviation for proficiency assessment (SDPA).

If the condition of Equation (5) is satisfied, it is interpreted as a “Warning signal”, additionally if the condition of Equation (6) is satisfied, it indicates the need to take some action—“Action signal”.

(b) **Score** *z* is obtained as follows:

(7) $$z=\frac{\left(x-X\right)}{\overset{⌢}{\sigma }}$$

With results interpretation is as follows:

(8) |z|≤2

(9) 2≤|z|≤3

(10) |z|≥3

If the condition of Equation (8) is satisfied, the comparison results are “Satisfactory”; Equation (9) generates a “Warning signal” and Equation (10) indicates the need to take some action—“Action signal”

(c) **Number** $E_{n}$, which is calculated as follows:
  (11) $$E_{n}=\frac{\left(x-X\right)}{\sqrt{U_{x}{}^{2}+U_{X}{}^{2}}}$$
  where:

$U_{x}{}^{2}$ is an estimate of the standard uncertainty from the result from the Participant,

$U_{X}{}^{2}$ is standard uncertainty from the reference assigned value.

With the interpretation of the results:

(12) $$\left|E_{n}\right|\leq 1$$

(13) $$\left|E_{n}\right|\geq 1$$

Equation (12) generates a “Satisfactory” result, while Equation (13) generates an “Unsatisfactory” result.

For the purpose of this research, the results for comparison were selected according to the rules of creation and cartographic presentation of the Digital Cadastral Map (DCM). The contents of the DCM consist of: point, line, and polygon features. Point features are categorized as points of the geodetic reference base and points that characterize the spatial detail. The results for the comparison of point features are GNSS coordinates that can be transformed into **bias** *D*. Linear features are lines, defined by a set of points (at least two) as well as a topographic label. The result for comparing line features is the length of the line that can be transformed into **Score** *z*. Polygon features define spatial entities with appropriate spatial attributes. The result for comparing polygon features is its polygon area that can be transformed into a **Number** $E_{n}$.

## 3. Research Results

**Figure 2:**
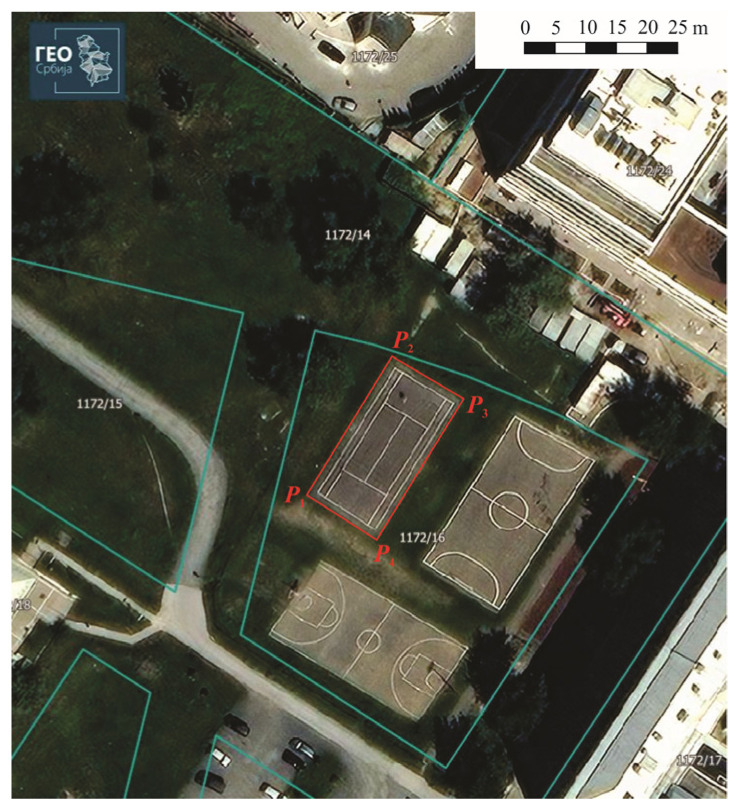
Test polygon.

The purpose of the InterGNSS Network comparison is to determine and validate the competence of three GNSS permanent networks in the Republic of Serbia. For the realization of the InterGNSS Network comparison, a Test polygon was created with four points: (Figure 2, downloaded from the GEOSRBIJA portal [28]) in the city of Belgrade. The points were stabilized with geodesic pins in the asphalt of the Tennis court (Test polygon), assuring their stability.

### 3.1. Assigned Value and Its Uncertainty in the InterGNSS Network Comparison

The assigned value should be determined in accordance with professional experience and the needs of the GNSS Network. To prepare this comparison, the following were analyzed:

- Directly determined values, i.e., coordinates (E, N, h) and
- Indirectly determined values, as functions of the coordinates $f\left(E_{i},\;N_{i},\;h_{i}\right)$, i=1,…n, i.e., distance (*S*) and polygon area (*P*) calculated from coordinates.

To determine the standard deviation and measurement uncertainty, we use the law of propagation of errors and combined standard uncertainty evaluation [23], which are well-known in the geodetic profession.

For the Test polygon and GNSS Network of Participants: AGROS; “Vekom-Net”; “GentooARS”, the following were calculated:

(a) **bias** *D:* for coordinates E, N,h in the ETRS89/UTM zone 34N system:
  (14) $$D_{E_{i}}=\left(E_{i}-E\right),\;D_{N_{i}}=\left(N_{i}-N\right),\;D_{h_{i}}=\left(h_{i}-h\right)$$
  for each of Participants, *i* = 1, 2, 3 (Chapter 3.2).
(b) **Score** *z*: as the distance (line length) between two points of the Test Polygon:
  (15) $$z=\frac{\left(s_{1,2}-S_{1,2}\right)}{\overset{⌢}{\sigma }}$$
  where:

*s*_1,2_ is measured line length/distance result from the Participant,

*S*_1,2_ is the assigned distance value of the line feature.

To calculate the standard deviation ($\overset{⌢}{\sigma }$) for the distance/line length, we start from the equation:

(16) $$S_{1,2}={\left[{\left(E_{2}-E_{1}\right)}^{2}+{\left(N_{2}-N_{1}\right)}^{2}\right]}^{1/2}$$

By applying the law of error propagation on Equation (16) as follows:

(17) $${\overset{⌢}{\sigma }}_{}^{2}={\left(\frac{\partial S}{\partial E_{2}}\right)}^{2}\cdot \sigma_{E_{2}}^{2}+{\left(\frac{\partial S}{\partial E_{1}}\right)}^{2}\cdot \sigma_{E_{1}}^{2}+{\left(\frac{\partial S}{\partial N_{2}}\right)}^{2}\cdot \sigma_{N_{2}}^{2}+{\left(\frac{\partial S}{\partial N_{1}}\right)}^{2}\cdot \sigma_{N_{1}}^{2}$$

is obtained:

(18) $$\begin{array}{l} \frac{\partial S}{\partial E_{2}}=\frac{1}{S}\left(E_{2}-E_{1}\right)\\ \frac{\partial S}{\partial E_{1}}=-1\cdot \frac{1}{S}\left(E_{2}-E_{1}\right)\\ \frac{\partial S}{\partial N_{2}}=\frac{1}{S}\left(N_{2}-N_{1}\right)\\ \frac{\partial S}{\partial N_{1}}=-1\cdot \frac{1}{S}\left(N_{2}-N_{1}\right) \end{array}$$

and equalizing the effects yields the following equation:

(19) $$\sigma_{E_{2}}^{2}=\sigma_{E_{1}}^{2}=\sigma_{N_{2}}^{2}=\sigma_{N_{1}}^{2}=\sigma_{0}^{2}$$

(20) $${\overset{⌢}{\sigma }}_{}^{}=\sqrt{2}\cdot \sigma_{0}^{},\;i.e.,\;{\overset{⌢}{\sigma }}_{}^{}=\sqrt{2}\cdot \sigma_{0}^{}$$

(c) **Number**$E_{n}$: is calculated as the polygon area of a Tennis court obtained as follows:
  (21) $$E_{n}=\frac{\left(p-P\right)}{\sqrt{U_{p}{}^{2}+U_{P}{}^{2}}}$$
  where:

*p* is measured polygon area result from the Participant,

*P* is the assigned polygon area value of the Tennis court,

$U_{p}{}^{2}$ is an estimate of the standard uncertainty from the result from the Participant,

$U_{P}{}^{2}$ is standard uncertainty from the assigned value.

To calculate the standard deviation of the polygon area of the Tennis court $P_{1,2,3,4}=f\left(E_{i},N_{i}\right)$, i=1,2,3,4, we start from the following:

(22) $$P_{1,2,3,4}=\frac{1}{2}\sum E_{n}\left(N_{n-1}-N_{n+1}\right)=\frac{1}{2}\sum \left[E_{1}\left(N_{4}-N_{2}\right)+E_{2}\left(N_{1}-N_{3}\right)+E_{3}\left(N_{2}-N_{4}\right)+E_{4}\left(N_{3}-N_{1}\right)\right]$$

By applying the law of propagation of errors:

(23) $${\overset{⌢}{\sigma }}_{}^{2}=\sum_{}\left[{\left(\frac{\partial P}{\partial E_{n}}\right)}^{2}\cdot \sigma_{E_{n}}^{2}+{\left(\frac{\partial P}{\partial N_{n}}\right)}^{2}\cdot \sigma_{N_{n}}^{2}\right],\;n=1,2,3,4$$

where:

(24) $$\frac{\partial P}{\partial E_{n}}=\frac{1}{2}\left(N_{n-1}-N_{n+1}\right);\;\frac{\partial P}{\partial N_{n}}=\frac{1}{2}\left(E_{n+1}-E_{n-1}\right)$$

is obtained:

(25) $${\overset{⌢}{\sigma }}^{2}=\begin{array}{l} {\left(\frac{1}{2}\left(N_{4}-N_{2}\right)\right)}^{2}\cdot \sigma_{E_{1}}^{2}+{\left(\frac{1}{2}\left(N_{1}-N_{3}\right)\right)}^{2}\cdot \sigma_{E_{2}}^{2}+{\left(\frac{1}{2}\left(N_{2}-N_{4}\right)\right)}^{2}\cdot \sigma_{E_{3}}^{2}+{\left(\frac{1}{2}\left(N_{3}-N_{1}\right)\right)}^{2}\cdot \sigma_{E_{4}}^{2}+\\ {\left(\frac{1}{2}\left(E_{2}-E_{4}\right)\right)}^{2}\cdot \sigma_{N_{1}}^{2}+{\left(\frac{1}{2}\left(E_{3}-E_{1}\right)\right)}^{2}\cdot \sigma_{N_{2}}^{2}+{\left(\frac{1}{2}\left(E_{4}-E_{2}\right)\right)}^{2}\cdot \sigma_{N_{3}}^{2}+{\left(\frac{1}{2}\left(E_{1}-E_{3}\right)\right)}^{2}\cdot \sigma_{N_{4}}^{2} \end{array}$$

By equalizing the impacts of all parameters in Equation (25): $\sigma_{E_{n}}^{2}=\sigma_{N_{n}}^{2}=\sigma_{0}^{2}$, for n=1,2,3,4 the following is obtained:

(26) $${\overset{⌢}{\sigma }}_{}^{2}={\left(\frac{1}{2}\right)}^{2}\cdot \sigma_{0}^{2}\left[\begin{array}{l} {\left(N_{4}-N_{2}\right)}^{2}+{\left(N_{1}-N_{3}\right)}^{2}+{\left(N_{2}-N_{4}\right)}^{2}+{\left(N_{3}-N_{1}\right)}^{2}+\\ {\left(E_{2}-E_{4}\right)}^{2}+{\left(E_{3}-E_{1}\right)}^{2}+{\left(E_{4}-E_{2}\right)}^{2}+{\left(E_{1}-E_{3}\right)}^{2} \end{array}\right]$$

(27) $${\overset{⌢}{\sigma }}_{}^{2}={\left(\frac{1}{2}\right)}^{2}\cdot \sigma_{0}^{2}\cdot 2\left[S_{2-4}^{2}+S_{1-3}^{2}\right]=\frac{\sigma_{0}^{2}}{2}\left[S_{2-4}^{2}+S_{1-3}^{2}\right]$$

### 3.2. The Results of Comparison

The results for comparison were achieved in the following measurements mode:

- Static GNSS mode obtained on Test polygon points independent of participating GNSS Networks; and
- GNSS RTK mode within each of participating GNSS Networks (AGROS; “Vekom-Net”; “GentooARS”).

After all planned measurements were finished at the Test polygon (Figure 2), the analysis of the obtained values and assessment of conformity for point (criterion **bias** *D*), line (criterion **Score** *z*) and polygon features (criterion **Number** $E_{n}$) were calculated.

**Figure 3:**
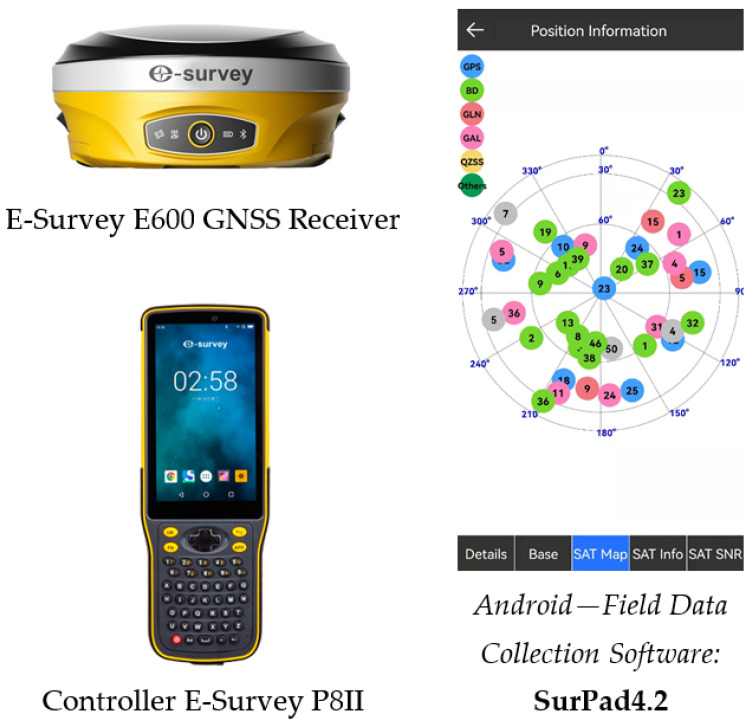
Measurement equipment.

**Figure 4:**
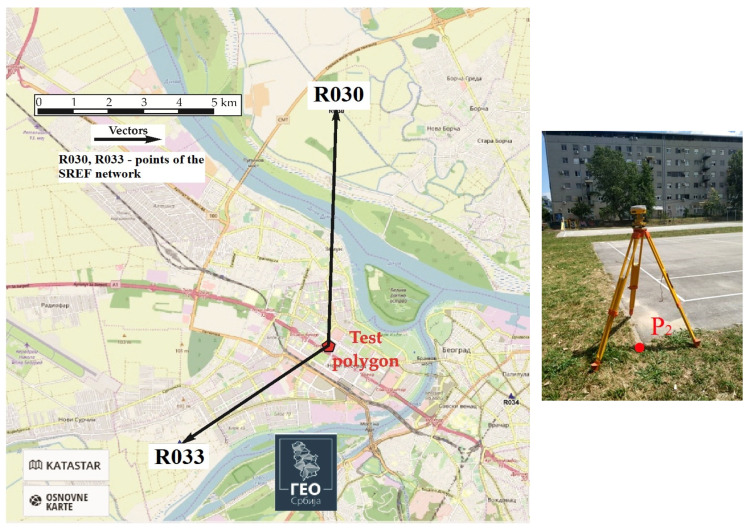
Measurements in the Test polygon—Static GNSS mode (point P_2_ of the Test polygon).

**Figure 5:**
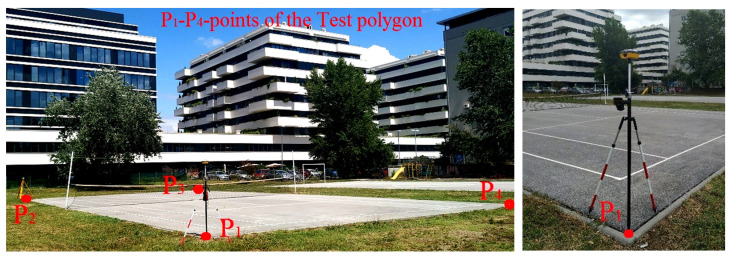
Measurements in the Test polygon—GNSS RTK mode (point P_1_ of the Test polygon).

For the Test polygon and each of the Participants, the following were calculated:

(a) - **bias** *D:* by using Equation (14) for each of the coordinates $D_{E_{i}}=\left(E_{i}-E\right)$, $D_{N_{i}}=\left(N_{i}-N\right)$, $D_{h_{i}}=\left(h_{i}-h\right)$
  - The GNSS static positioning mode implies that the assigned value and its uncertainty are determined. Each point of the Test polygon was determined with two vectors according to the points of the SREF network [14] in one session lasting 1 h, with GNSS E-Survey E600 receiver (eSurvey GNSS, Shanghai, China) (Figure 3). The GNSS observations were processed in the Topcon Tools software (Topcon, Tokyo, Japan) [29]. Static GNSS mode measurements are shown in Figure 4.
  - The GNSS RTK mode allows the calculation of **bias** *D* for each Participant, i=1, 2, 3. When checking the measurement uncertainty between the three GNSS networks, the measurements were performed in three phases, using the software supplied by the GNSS equipment manufacturer, in accordance with the manufacturer’s instructions. The GNSS measurement results are dependent on the layout of the GNSS satellites, the state of the ionosphere and troposphere, and the environment at the time of the measurements. In order to lower listed impacts on GNSS RTK measurements, the observations from Participants were determined in the shortest possible period using the same GNSS equipment: E-Survey E600 receiver, E-Survey P8II controller, and SurPad4.2 software (eSurvey GNSS) [30] (Figure 3). GNSS RTK mode measurements are shown in Figure 5.

GNSS RTK mode measurements were done through the following phases:

- In the first phase, the GNSS receiver was connected to the first GNSS Network of Participants in the comparison—Participant 1: AGROS.
- In the second phase, the GNSS receiver was connected to the second GNSS Network of Participants in the comparison—Participant 2: “Vekom Net”,
- In the third phase, the GNSS receiver was connected to Participant 3: “GentooARS”.

Five series of GNSS measurements/observations were carried out at the Test polygon points in each of the GNSS Networks of Participants. An interval between two successive GNSS measurements was approximately 5 min; the time required for one series/GNSS session was about 25 min, and measurements were repeated according to the same model for each GNSS Network of Participants.

The standard deviation for proficiency assessment (SDPA) ($\overset{⌢}{\sigma }$) is specified by the manufacturer of GNSS equipment ($\overset{⌢}{\sigma }={\overset{⌢}{\sigma }}_{E}={\overset{⌢}{\sigma }}_{N}={\overset{⌢}{\sigma }}_{E,N}/\sqrt{2}$) so that the GNSS measurement results can be interpreted according to the criteria specified in Equation (5). Corresponding value $\sigma_{E,N}$, $\sigma_{h}$ specified by the manufacturer of E-Survey E600 are: $\sigma_{E,N}=8\;\mathrm{mm}$, $\sigma_{h}=15\;\mathrm{mm}$, i.e., ${\overset{⌢}{\sigma }}_{E}={\overset{⌢}{\sigma }}_{N}=8/\sqrt{2}=5.7\;\mathrm{mm}$.

Characteristic values for **bias** *D* for the Test polygon points are shown in Table 2, Table 3 and Table 4. Bias D values greater than 17.1 mm for coordinates *E* and *N* indicate the need to take an “Action signal” or to limit geodetic works in which coordinates are compared by coordinate axes.

(b) **Score** *z* is calculated as a distance (line length) between points P_1_ and P_2_ ${\left(E,\;N\right)}_{1,2}$ of the Tennis court. Characteristic values for **score** *z* are shown in Table 5.

If $\sigma_{0}=5.7\;\mathrm{mm}$, then it follows that the standard deviation for short distances is $\overset{⌢}{\sigma }=\sqrt{2}\cdot \sigma_{0}^{}=8.0\;\mathrm{mm}$.

(c) **Number** $E_{n}$ was calculated as the polygon area of a Tennis court.

To calculate the standard measurement uncertainty of the polygon area, it is necessary to calculate the extended measurement uncertainty of the coordinate function. Measurement uncertainty for 2D and 1D coordinates were analyzed according to the ISO 17123-8:2015 Standard. Combined uncertainty on the horizontal coordinate system for evaluating the results can be determined according to [17].

Combined uncertainty on the horizontal coordinate system (ETRS89/UTM zone 34N) is described as:

(28) $$u_{EN}=\sqrt{u_{\mathrm{ISO}-\mathrm{GNSS}-EN}^{2}+{\left[h_{a}\tan\left(u_{\mathrm{bub}}\right)\right]}^{2}+2u_{\mathrm{disp}}^{2}+u_{c}^{2}+u_{dE}^{2}+u_{dN}^{2}+u_{\mathrm{tr}}^{2}}$$

Combined uncertainty on the vertical coordinate system is described as:

(29) $$u_{h}=\sqrt{u_{\mathrm{ISO}-\mathrm{GNSS}-h}^{2}+u_{\mathrm{disp}}^{2}+u_{ha}^{2}+u_{hS}^{2}+u_{dh}^{2}+u_{\mathrm{ge}}^{2}}$$

Expanded uncertainty, with coverage factor k = 2,

(30) $$\begin{array}{l} U_{EN}=2\cdot u_{EN}\\ U_{h}=2\cdot u_{h} \end{array}$$

For the values from Table 6, the values for Equations (28) and (29) are obtained as follows:

$$u_{EN}=\sqrt{8.0^{2}+3.49^{2}+2\cdot 0.29^{2}+1^{2}+1^{2}+1^{2}+1^{2}}=8.96\;\mathrm{mm}$$

And per coordinates:

$$u_{E}=u_{N}=u_{EN}/\sqrt{2}=8.96/\sqrt{2}=6.33\;\mathrm{mm}$$

$$u_{h}=\sqrt{15^{2}+0.29^{2}+1^{2}+0+2^{2}+0.56^{2}}=15.18\;\mathrm{mm}.$$

Values for the Equation (30) are:

$$U_{EN}=2\cdot u_{EN}=2\cdot 8.96=17.92\;\mathrm{mm}$$

$$U_{h}=2\cdot u_{h}=2\cdot 15.18=30.36\;\mathrm{mm}.$$

By substituting the standard deviation with the measurement uncertainty and considering that in the Test polygon, it stands true that $S_{2-4}^{}=S_{1-3}^{}=S$, from Equation (27) is obtained that:

(31) $$u_{p}=\frac{u_{EN}^{2}}{2}\left[2\cdot S^{2}\right]$$

it follows that the standard measurement uncertainty of the polygon area of the Tennis court is:

(32) $$u_{p}=u_{EN}^{}\cdot S$$

where: $u_{E_{n}}^{}=u_{N_{n}}^{}=u_{EN}=6.33\;\mathrm{mm}$, *S* = 29.1 m, $u_{p}=u_{EN}\cdot S=0{.184\;m}^{2}$.

Expanded uncertainty, with coverage factor k = 2,

(33) $$U_{p}=2\cdot u_{p}$$

is obtained:

(34) $$U_{p}=2\cdot 0.184=0.367{\;m}^{2}.$$

The assigned value for the Test polygon area was calculated from the coordinates determined from static GNSS measurement mode using Equation (22). The measurement uncertainty of the position of the points in Test polygon obtained by the static GNSS mode was calculated using Equation (28). In Equation (28), the value $u_{\mathrm{ISO}\text{-}\mathrm{GNSS}\text{-}EN}^{2}$ is replaced by the standard deviations of the positions of the points $\sigma_{EN}$, obtained from the adjustment of static GNSS mode measurements. The other values are the same and are taken from Table 6. From the static GNSS mode the positional error $\sigma_{EN}=2.8\;\mathrm{mm}$ is obtained, from which it follows that the expanded uncertainty is: $U_{NE}=9.75\;\mathrm{mm}$, accordingly $U_{P}=0.200{\;m}^{2}$.

## 4. Discussion

GNSS RTK measurements at the Test polygon were performed in accordance with the rules prescribed by the “Rulebook on the application of Global Navigation Satellite System technology in the areas of State Survey and Cadastre” [31] adopted by the RGA. For the purpose of determining the GNSS coordinates of the ground points in GNSS RTK mode, this Rulebook prescribes that minimum GNSS observation time must be 5 s with a registration interval of one second; PDOP (Position Dilution of Precision) during GNSS measurements must be less than 8 and determination of GNSS coordinates must be done using two-frequency GNSS receivers of geodetic type. While performing GNSS RTK measurement at the Test polygon the cut-off angle of satellite signals was 15°.

The Test polygon (Figure 2) is situated in an open area where there is no possibility of GNSS signal reflection. GNSS system (Figure 3) and the applied commercial software do not have the ability to change data on atmospheric conditions, and therefore do not have the ability to calculate the impact of the ionosphere and troposphere while performing GNSS RTK measurements. It should be noted that most of the commercial software delivered by manufacturers of GNSS devices for geodetic purposes does not have this ability. The parameters of the atmosphere should be taken into account and administered by GNSS correction distributors, i.e., owner of the GNSS Network (AGROS; “Vekom-Net”; “Gen-tooARS”). However, since all GNSS RTK measurements (in all three GNSS Networks) at the Test polygon were performed in a short period of time (total measurements lasted app. 90 min), it can be concluded that the external influences were the same for all measurements and that they affected the measurement results in the same way.

The intention of the authors of the paper was to test the GNSS data collection methodology that is most used in geodetic practice. For that reason, the GNSS RTK measurements were performed according to the aforementioned Rulebook on the application of Global Navigation Satellite System technology in the areas of State Survey and Cadastre [31].

For control measurements (Figure 4) in static GNSS mode, commercial software Topcon Tools was used, because the lengths of the vectors to the SREF network points were less than 10 km and there was no need to use scientific software, such as: Bernese GNSS Software; GNSS-Inferred Positioning System and Orbit Analysis Simulation Software (GIPSY-OASIS); etc. Commercial software Topcon Tools allowed users to choose a troposphere model (in this case the Classical Hopfield model was used) and a classical meteorological model with standard atmospheric values (used in this case) or measured atmospheric values that can be entered for GNSS vector processing. Based on the information available to the authors, the users of each GNSS vector processing software for geodetic purposes, rarely or never measure the atmospheric values to calculate their effect on GNSS measurement.

Homogeneity testing in the direction of the coordinate axes: E, N, h (Table 2, Table 3 and Table 4) indicates that an “Action signal” should be taken. While performing measurements in the field of engineering geodesy, a user of the GNSS RTK Network must be aware of the fact that they cannot use measurements made in different epochs and different GNSS Networks and draw conclusions about deformation analysis through coordinates (E, N, h). Confirmation of that claim results from deviations that do not satisfy evaluation criteria: $2\cdot \hat{\sigma }<D<-2\cdot \hat{\sigma }$ or $3\cdot \hat{\sigma }<D<-3\cdot \hat{\sigma }$. However, when the homogeneity was analyzed through derived dimensions (length or area), the evaluation “Satisfactory” was achieved according to the criteria: Score z and Number $E_{n}$ (Table 6 and Table 7).

The deviations noted at the Test polygon, can be caused by the corrections sent to the GNSS receivers from the GNSS Network, i.e., AGROS; “Vekom Net” and “GentooARS” while performing GNSS RTK measurements. Some studies “show unresolved issues in the utilization of position estimates in geographical reference frame for GNSS positioning performance assessment. Those lead to a recommendation for GNSS positioning performance assessment based on original WGS84-based GNSS position” [32].

## 5. Conclusions

Standard methods for calibrating geodetic instruments have been published by the Technical Committee: ISO/TC 172/SC 6 Geodetic and surveying instruments. Calibration of geodetic instruments is performed in accredited metrological laboratories. The calibration result is recorded in a document called Calibration Certificate. All the instruments and equipment that were used in this research have a Calibration Certificate issued by Laboratory 02-077, which is accredited according to the ISO/IEC 17025 Standard.

“Based on activities such as technical standardization, metrology, testing, calibration, certification, and accreditation—which are jointly known as the national quality infrastructure—it is possible to provide assurance those products, processes, people, and management systems meet standard technical requirements.” [33]. “Standard of ISO 17123 specifies field procedures for adoption when determining and evaluating the uncertainty of measurement results obtained by geodetic instruments and their ancillary equipment, when used in building and surveying measuring tasks. Primarily, these tests are intended to be field verifications of the suitability of a particular instrument for the immediate task” [33]. “The uncertainty evaluation of survey measurements is a daily and essential task in any surveying work. The result of a measurement is, in fact, only complete when accompanied by a statement of its uncertainty” [34,35,36].

Starting from standardized principles and guidelines, the authors of this paper, based on decades of experience, presented the possibilities of checking the traceability of measurement results obtained in different GNSS Networks. This procedure is based on the measurement of coordinates in the Test polygon with known reference values. The standard deviations of point coordinates are defined in the user’s software under conditions for monitoring and control and depend on the accuracy of the GNSS receiver in the RTK mode. The behavior of the GNSS network depends on the characteristics of the embedded software for its work and control. As shown in this research a comprehensive analysis necessarily requires a comparison of the coordinates obtained in different GNSS Networks licensed for work on a territory of a country. The InterGNSS Network comparison is a comparative analysis of a group of GNSS networks that are operational over the territory of one country, and it must be a well-established practice for monitoring of work of GNSS Networks in geodetic science and practice.

Establishing quantitative metrics for their comparison and testing is not an easy task. In order for the InterGNSS Network comparison to be recognized, it is necessary to set a number of requirements, including the prior publication of all criteria for a “Satisfactory” result or an “Unsatisfactory” result.

The obtained results in this research indicate the feasibility of establishing quantitative comparisons between GNSS Networks for analyzing and testing the homogeneity of coordinates calculated by different GNSS Networks.

In October 2019, the Director of the RGA issued Instructions for reviewing studies where the GNSS measurement method is applied in the cadastre maintenance procedure [37]. The next step should be improving the quality and confidence in the results obtained by GNSS Network, as well as defining a regular period and method of comparison among different GNSS Networks. It should be prescribed situations for extraordinary checks and controls of works of GNSS Networks, too. The authors of this paper propose that RGA, as a governmental institution under whose supervision GNSS Networks are, should take into consideration the need for regular testing of homogeneity of coordinates of various permanent GNSS reference stations networks of the Republic of Serbia according to the common requirements for proving competence presented in this research, and prescribe it through a legal framework.

## Figures and Tables

**Table 1:** Characteristic deviation of the GNSS coordinates from their mean values per measurement’s day.

Day		Δ*E* (m)	Δ*N* (m)	Δ*h* (m)
1	max.	0.049	0.029	0.082
	min.	−0.036	−0.025	−0.081
2	max.	0.162	0.015	0.079
	min.	−0.077	−0.020	−0.054
3	max.	0.160	**0.400**	0.490
	min.	**−0.356**	−0.191	**−0.644**

**Table 2:** **Bias** *D* for the coordinate *E* (*i* = AGROS; “Vekom Net”; “GentooARS”).

Pt.	Assigned Value	Results from Participant		Evaluation
	*E*	$E_{i}$	$D_{E_{i}}$	$3\cdot \hat{\sigma }<D<-3\cdot \hat{\sigma }$
	(m)	(m)	(mm)	(mm)
1	4,963,054.011	4,963,054.004	0.1	17.1
		4,963,054.021	**17.7**	17.1
		4,963,054.008	5.5	17.1
2	4,963,075.926	4,963,075.923	**18.6**	17.1
		4,963,075.931	**27.2**	17.1
		4,963,075.924	**20.2**	17.1
3	4,963,069.143	4,963,069.146	12.8	17.1
		4,963,069.150	16.6	17.1
		4,963,069.132	−0.8	17.1
4	4,963,046.934	4,963,046.925	1.2	17.1
		4,963,046.943	**18.8**	17.1
		4,963,046.934	9.9	17.1

**Table 3:** **Bias** *D* for the coordinate *N* (*i* = AGROS; “Vekom Net”; “GentooARS”).

Pt.	Assigned Value	Results from Participant		Evaluation
	*N*	$N_{i}$	$D_{N_{i}}$	$3\cdot \hat{\sigma }<D<-3\cdot \hat{\sigma }$
	(m)	(m)	(mm)	(mm)
1	453,415.431	453,415.431	**19.1**	17.1
		453,415.442	**30.5**	17.1
		453,415.421	9.1	17.1
2	453,428.955	453,428.953	10.4	17.1
		453,428.970	**27.0**	17.1
		453,428.941	−2.2	17.1
3	453,440.144	453,440.154	**21.0**	17.1
		453,440.149	16.0	17.1
		453,440.130	−3.4	17.1
4	453,426.414	453,426.422	**21.8**	17.1
		453,426.418	**18.0**	17.1
		453,426.403	2.6	17.1

**Table 4:** **Bias** *D* for the height *h* (*i* = AGROS; “Vekom Net”; “GentooARS”).

Pt.	Assigned Value	Results from Participant		Evaluation
	*H*	$h_{i}$	$D_{h_{i}}$	$3\cdot \hat{\sigma }<D<-3\cdot \hat{\sigma }$
	(m)	(m)	(mm)	(mm)
1	119.347	119.360	28.2	45.0
		119.340	8.4	45.0
		119.340	7.8	45.0
2	119.174	119.192	43.8	45.0
		119.174	26.4	45.0
		119.155	7.0	45.0
3	119.147	119.154	32.4	45.0
		119.151	28.8	45.0
		119.135	13.4	45.0
4	119.291	119.294	28.0	45.0
		119.299	32.8	45.0
		119.279	13.4	45.0

**Table 5:** **Score***z*, for the distance *S*_1-2_ (*i* = AGROS; “Vekom Net”; “GentooARS”).

Assigned Value	Results from Participant				Evaluation
*s*	$S_{i}$	$s-S_{i}$	$\overset{⌢}{\sigma }$	*z*	|z|≤2
(m)	(m)		(mm)		
25.7436	25.7548	−11.17	8.0	1.40	“satisfactory”
	25.7481	−4.46	8.0	0.56	“satisfactory”
	25.7510	−7.42	8.0	0.93	“satisfactory”

**Table 6:** Typical influence quantities of the GNSS (RTK) [17]—Taken over from ISO 17123-8:2015, Table 3.

Sources of Uncertainty	Symbol	Evaluation/Distribution	Sources of Uncertainty
**I. Result of measurement**			
Standard uncertainty of *EN*-coordinates Standard uncertainty of *h*-coordinates	$u_{\mathrm{ISO}\text{-}\mathrm{GNSS}\text{-}EN}$ $u_{\mathrm{ISO}\text{-}\mathrm{GNSS}\text{-}h}$	Normal/Type A	$\mathrm{corresponding}\;\mathrm{value}\;\sigma_{E,N},\;\sigma_{h}$ stated by the manufacturer $u_{\mathrm{ISO}\text{-}\mathrm{GNSS}\text{-}EN}=\sigma_{E,N}=8\;\mathrm{mm}$ $u_{\mathrm{ISO}\text{-}\mathrm{GNSS}\text{-}h}=\sigma_{h}=15\;\mathrm{mm}$
**II. Relevant sources of the GNSS Receiver**			
Sensitivity of the tubular level on tribrach	$u_{\mathrm{bub}}$	Specified by the manufacturer/ Type B	For a level sensitivity of 8′, $h_{a}\tan\left(u_{\mathrm{bub}}\right)=3.49\;\mathrm{mm}$
The standard uncertainty of the minimum display digit	$u_{\mathrm{disp}}$	Rectangular/Type B	$u_{\mathrm{disp}E}=u_{\mathrm{disp}N}=u_{\mathrm{disp}h}$ $=0.5/\sqrt{3}=0.29\;\mathrm{mm}$
**III. Error pattern from the setting of the instruments**			
The standard uncertainty of the centering	$u_{c}$	Normal/Type B	1 mm
The standard uncertainty of the antenna height	$u_{ha}$	Normal/Type B	1 mm
Stability of a tripod height (ISO 12858-2:1999)	$u_{hS}$	Rectangular/Type B	The stability of the height is estimated within 0.05 mm, which is neglectable from the budget.
The standard uncertainties of the antenna phase center	$u_{dE}$, $u_{dN}$, $u_{dh}$	Normal/Type B	are based on general knowledge $u_{dE}=u_{dN}=1\;\mathrm{mm}$ $u_{dh}=2\;\mathrm{mm}$
Multi path; Clock in the GNSS receiver or satellite; Orbit of the satellite; Ionospheric delay; Tropospheric delay		Not considered here	/
**IV. Mathematical modeling**			
Transformation	$u_{\mathrm{tr}}$	Normal/Type A	1 mm
The standard uncertainty of the geoid undulation	$u_{ge}$	Rectangular/Type B	When the baseline is 20 m, geoid height difference between the base and rover is 1.95 mm or less. $u_{ge}=1.94/\left(2\cdot \sqrt{3}\right)=0.56\;\mathrm{mm}$

**Table 7:** **Number** $E_{n}$, for polygon area (*i* = AGROS; “Vekom Net”; “GentooARS”).

*P*	$U_{P}$	*p*	$U_{p}$	p−P	$E_{n}$	Evaluation
(m^2^)	(mm^2^)	(m^2^)	(mm^2^)	(m^2^)		
338.977	200.37	339.290	367.33	312.89	0.75	“satisfactory”
		338.856	367.15	−120.95	0.29	“satisfactory”
		338.968	367.16	−8.98	0.02	“satisfactory”

## Data Availability

Not applicable.
